# A Randomized Double-Blind Trial of the Effect of Liupao Tea on Metabolic Parameters, Body Composition, and Gut Microbiota in Adults with Metabolic Syndrome

**DOI:** 10.3390/nu17142371

**Published:** 2025-07-19

**Authors:** Yuyang Wang, Qiang Hu, Qiliu Jiang, Jiamin Jiang, Biandi Li, Defu Ma

**Affiliations:** 1 Department of Social Medicine and Health Education, School of Public Health, Peking University Health Science Center, Beijing 100191, China; 2 Zhaoping Tea Science and Technology Courtyard, Zhaoping, Hezhou 546800, China

**Keywords:** liupao tea, metabolic syndrome, lipid metabolism, body composition, gut microbiota, children

## Abstract

**Background**: Metabolic syndrome (MetS) represents a significant global health challenge. Liupao tea (LPT), a post-fermented dark tea, has shown potential metabolic benefits, but clinical evidence remains limited. **Objectives**: This study aimed to investigate the effects of LPT with varying aging durations on clinical parameters, body composition and gut microbiota in individuals with MetS. **Methods**: In a randomized, double-blind trial, patients with MetS were randomly assigned to intervention groups, receiving 6 g/day of LPT aged for 1, 4, 7, or 10 years, respectively, over a 90-day intervention period. Blood pressure, lipid and glucose levels, body weight, body composition, and gut microbiota were assessed at baseline and post-intervention. **Results**: A total of 71 participants, with a mean age of 53.5 years, were included. At the final assessment, significant reductions in both systolic and diastolic blood pressure were observed in the 10-year-aged groups (*p* < 0.05). In terms of lipid profiles, the 1-year-aged group showed a significant decrease in total cholesterol (TC), while low-density lipoprotein cholesterol (LDL-C) levels significantly decreased in the 1-, 4-, 7-, and 10-year-aged groups (*p* < 0.05). All intervention groups showed significant reductions in body weight, body fat mass (BFM), along with an increase in muscle mass (MM) (*p* < 0.05). A decrease in the Firmicutes/Bacteroides (F/B) ratio was observed in the 10-year-aged group. No significant differences in clinical parameters or body composition regulation were observed between groups with varying aging durations (*p* > 0.05). **Conclusions**: LPT intervention effectively improves metabolic health and modulates gut microbiota in MetS patients, irrespective of aging duration. These findings support LPT as a functional beverage for the management of MetS.

## 1. Introduction

Metabolic syndrome (MetS) refers to a cluster of clinical conditions associated with multiple high-risk metabolic factors or diseases. The World Health Organization (WHO) defines MetS as a pathological state characterized by the clustering of chronic metabolic diseases. It is also known as Syndrome X [1]. The global prevalence of MetS is rising, driven by urbanization, sedentary lifestyles, and dietary changes [2]. In China, the overall prevalence of MetS is 22.0%, with 23.6% in females and 21.0% in males. Urban areas report a prevalence of 23.5%, while rural areas have a slightly lower rate at 20.3% [3]. Patients with MetS tend to experience higher medical expenditures, more frequent hospitalizations, and higher utilization rates of outpatient and physician services [4]. Compared with individuals without MetS, those affected by the syndrome incur annual health costs approximately three times higher, with costs increasing as the number of MetS components rises [5].

Growing evidence suggests that phytochemicals derived from natural plants have beneficial effects on improving MetS [6]. As a natural functional beverage, tea has shown notable promise in the field of metabolic health [7]. Numerous large-scale epidemiological studies have demonstrated a significant negative correlation between the consumption of black tea and green tea and the risk of MetS [8,9,10]. For instance, drinking more than five cups of tea per day is associated with a 14% reduction in the risk of MetS [11]. Furthermore, a large cohort study from the UK Biobank revealed that higher tea intake is inversely related to all-cause and cancer mortality in MetS patients, with daily consumption of ≥4 cups of tea reducing cardiovascular disease mortality by 11% [12]. A cross-sectional analysis of US adults also showed that hot tea consumption was significantly associated with lower body mass index (BMI), smaller waist circumference (WC), lower fasting blood glucose (FBG) levels in women, and higher high-density lipoprotein cholesterol (HDL-C) levels in men [13]. A systematic review by Marventano et al. synthesizing observational studies explicitly suggested that higher tea consumption reduces the odds of developing MetS by 17% [14].

Dark tea, in particular, holds greater potential for metabolic intervention compared to non-fermented teas due to its unique post-fermentation process. During the pile-fermentation process, polyphenol oxidase and microorganisms work synergistically to promote the conversion of catechins into active ingredients such as theabrownin and tea polysaccharides, endowing it with significant lipid-lowering, anti-inflammatory and prebiotic properties [15,16,17]. Liupao tea (LPT), a variety of dark tea, due to the high content of polyphenols, theabrownin and other bioactive compounds, has demonstrated notable anti-diabetic properties [18]. Wu et al. found that the extract of LPT can significantly reduce the body weight of obese mice fed a high-fat diet (HFD) and significantly alleviate liver damage and fat accumulation [19]. However, evidence from population-based intervention studies on LPT’s effects is still limited, especially concerning its role in regulating clinical parameters and gut microbiota in MetS patients.

Therefore, the purpose of this study is to explore the effects of LPT with different aging years on biochemical markers, body weight, body composition and gut microbiota in MetS patients; clarify the metabolic improvement efficacy of LPT as a natural plant functional beverage; and provide new evidence for the daily dietary intervention of patients with MetS and the health value of LPT.

## 2. Materials and Methods

### 2.1. Study Design

**Figure 1:**
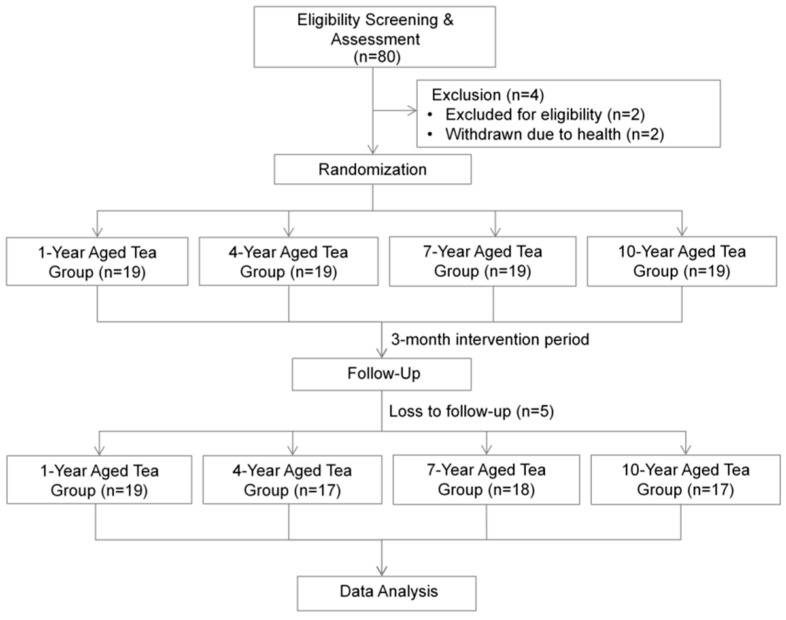
Study flowchart outlining the sequential steps from participant recruitment through to statistical data analysis.

This study employed a randomized, double-blind, longitudinal experimental design. It was approved by the Biomedical Ethics Committee of Peking University (IRB00001052-23203) and registered with the Chinese Clinical Trial Registry (Registration number: ChiCTR2400089348). Figure 1 illustrates the process from subject recruitment to data analysis.

### 2.2. Study Population

The recruitment of participants began on 1 April 2024 and concluded on 30 April 2024. Posters were displayed on the public bulletin boards of community streets in Haidian District, Beijing, to recruit trial subjects.

The inclusion criteria of this study are as follows: (1) age between 18 and 70 years old; (2) participants diagnosed with MetS. According to the Chinese Guidelines for the Prevention and Treatment of Type 2 Diabetes (2022 edition) [20], MetS can be diagnosed if 3 or more of the following 5 diagnostic criteria are met: ① WC > 90 cm in men and >85 cm in women; ② FBG > 6.1 mmol/L or blood glucose 2 h after glucose loading > 7.8 mmol/L, and/or previously diagnosed with diabetes; ③ systolic blood pressure (SBP) > 130 mmHg and/or diastolic blood pressure (DBP) > 85 mmHg, and/or previously diagnosed with hypertension; ④ triglyceride (TG) > 1.7 mmol/L; ⑤ HDL-C < 1.04 mmol/L. The participants understood the main content of this study, voluntarily participated in the study, and signed the informed consent form.

The exclusion criteria of this study include the following: (1) those with secondary abnormalities in blood glucose, blood lipids and blood pressure caused by nephrotic syndrome, hypothyroidism, liver diseases, etc.; (2) those with abnormal blood glucose and blood lipids caused by medications; (3) those with SBP > 180 mmHg or DBP > 110 mmHg after treatment; (4) those with FBG > 11.1 mmol/L after treatment with oral medications; (5) those with total cholesterol (TC) > 6.22 mmol/L, or low-density lipoprotein cholesterol (LDL-C) > 4.92 mmol/L, or TG > 3.39 mmol/L after treatment with oral medications; (6) those suffering from severe mental diseases, malignant tumors, severe heart diseases, blood system diseases, severe trauma, etc.; (7) pregnant or lactating women, or those planning to get pregnant during the trial period; (8) those with allergic constitution or those who may be allergic to the ingredients in tea; (9) special dietary habits, such as vegetarianism or ketogenic diet; (10) those who cannot cooperate for a long time or cannot follow the research requirements. All participants signed the informed consent form under the guidance of the researchers.

### 2.3. Sample Size Estimation

The estimation of the sample size was informed by a previous study conducted by Chu et al., which reported a baseline BMI of 28.67 ± 2.91 kg/m^2^ and a post-intervention BMI of 26.86 ± 3.17 kg/m^2^ [21]. A two-tailed test was employed, with the significance level set at α = 0.05 and the statistical power (1 − β) at 80%. The required sample size was computed using G*Power software (Version 3.1.9.7) [22], yielding a minimum of 16 participants per group. To accommodate a potential dropout rate of 10%, the target enrollment was increased to 17 individuals per group, resulting in a total of 68 participants. Additionally, a post hoc power analysis was carried out to validate the adequacy of the achieved sample size.

### 2.4. Randomization and Blinding

Participants were randomly allocated to four groups according to the aging durations of LPT, which were 1 year, 4 years, 7 years, and 10 years, respectively. Randomization was achieved using simple randomization, with a random number sequence generated by R software (version 4.1.3). This randomization process was conducted by professional statisticians who were not involved in the study. After informed consent was obtained, participants were assigned to their respective groups by opening sealed envelopes containing the random number sequence and group allocation. Each group received 6 g of LPT per day, with the aging duration varying according to the group. A double-blind design was adopted, meaning neither the participants nor the research team were aware of the group allocations. Participants were asked to collect pre-encapsulated tea samples 2 days before the start of the intervention and on days 28 and 58 during the intervention period.

### 2.5. Intervention and Adherence Monitoring

During the 90-day intervention phase, participants were required to follow a daily intervention protocol of consuming 6 g (one sachet) of LPT. Tea was brewed using a standardized method, where 250 mL of boiling water was poured into a 300 mL teapot, and the tea was steeped for 30–45 s before consumption. The tea bag could be re-steeped until the tea liquor noticeably faded. Participants were advised to consume the tea 1 to 2 h after breakfast or lunch and to finish it before 3 p.m. to avoid interference with sleep quality. Figure S1 shows the changes in the main water-soluble components of LPT with different aging years. The samples used in the experiment were provided free of charge by Guangxi Zhaoping County General Mountain Agricultural Technology Co., Ltd. (Hezhou, China)

To ensure adherence, an online WeChat group was created where participants uploaded daily photos of their tea consumption and maintained personal tea-drinking diaries. Researchers sent reminders to participants who missed their tea intake. Participants experiencing adverse effects were instructed to stop consuming tea and contact the research team. Non-compliant participants or those who were unreachable for three consecutive days were withdrawn from the study, with reasons for withdrawal documented. Participants were asked to maintain their usual diet, exercise, and routines during the study. Daily follow-ups via WeChat were conducted to monitor adherence and document any non-compliance or adverse events.

### 2.6. Questionnaire Survey and Clinical Parameter Measurement

At baseline, basic demographic information of participants was collected via questionnaire. The Visual Analogue Scale (VAS) was used to investigate the appetite of patients in the past week. The dietary status of patients was evaluated mainly from five aspects: hunger before meals, fullness after meals, intensity of appetite, amount of food intake, and mood during meals. The score range for each aspect is 0–100 points, with a total score of 500 points. The higher the score, the better the appetite of the patient.

On the testing day, fasting venous blood was drawn at around 8:00 a.m. following a minimum of 8 h of overnight fasting. The collected samples were placed into centrifuge tubes, processed by centrifugation, and subsequently aliquoted and stored at −80 °C in ultra-low-temperature freezers until further analysis. FBG levels were assessed using the hexokinase-based method, while glycated hemoglobin (HbA1c) was measured via ion-exchange chromatography. Fasting insulin (INS) concentrations were determined using a chemiluminescent immunoassay. Lipid profiles—including TC, TG, HDL-C, LDL-C, apolipoprotein A1 (APOA1), and apolipoprotein B (APOB)—were analyzed using standard enzymatic techniques. In addition, to assess the safety of the LPT intervention, several hepatic and muscle-related biomarkers were examined, including total bilirubin (T-BIL), alanine aminotransferase (ALT), aspartate aminotransferase (AST), alkaline phosphatase (ALP), creatine kinase (CK), and lactate dehydrogenase (LDH). All biochemical analyses were conducted in a certified clinical laboratory.

### 2.7. Body Composition Measurement

WC was measured with participants standing upright, relaxed, and arms naturally hanging. The measuring tape was placed 1 cm above the navel, and measurements were repeated twice. The average of the two measurements was recorded. Body composition was assessed using the IOI 353 analyzer (Jawon Medical Co., Ltd., Seoul, Republic of Korea), which was calibrated prior to testing to ensure measurement accuracy. During the procedure, participants wore light clothing, stood barefoot on the device’s metal footplates, and gripped the hand electrodes with both hands—ensuring contact with the thumb and all fingers—while maintaining relaxed, extended arms. Once the measurement was complete, participants stepped off the device. This analyzer utilizes bioelectrical impedance analysis (BIA) to estimate key physiological indicators, including body weight, body fat mass (BFM), skeletal muscle mass (MM), body fat percentage (BFP), lean body mass (LBM), and visceral fat area (VFA).

### 2.8. DNA Extraction and Amplification Library Construction

Fecal samples were stored at −80 °C prior to DNA extraction. Genomic DNA was extracted using a sodium dodecyl sulfate-based method, with strict quality control procedures in place to ensure it met the criteria for subsequent amplification and sequencing steps. The bacterial 16S rRNA gene’s V3–V4 hypervariable regions were targeted using the primer pair 515F and 806R. Each polymerase chain reaction (PCR) contained 15 μL of Phusion^®^ High-Fidelity PCR Master Mix (New England Biolabs, Ipswich, MA, USA), 0.2 μM of each primer, and 10 ng of template DNA. The amplification products were assessed using 2% agarose gel electrophoresis. PCR products were then cleaned with magnetic beads, and DNA fragments of suitable length were selected. Equimolar quantities from each sample were pooled to construct the sequencing library, which was subsequently sequenced using 250 bp paired-end reads on the Illumina NovaSeq platform (Illumina, Inc., San Diego, CA, USA).

### 2.9. Bioinformatics Analysis and Diversity Analysis

The sequencing data were initially demultiplexed according to sample-specific barcodes and primer sequences. Paired-end reads were merged at their overlapping regions using FLASH software (v1.2.11, https://ccb.jhu.edu/software/FLASH/ (accessed on 15 January 2025)) to generate raw sequence tags. Sequence quality control was conducted using fastp (v0.23.1), which eliminated reads of low quality, as well as adapter contaminants and primer dimers, thereby generating high-confidence clean tags. To remove chimeric sequences, comparisons were made against the Silva reference database (v2.15.0) using the Vsearch algorithm, yielding a refined set of valid sequence tags. Further denoising was implemented through either the DADA2 pipeline or the deblur plugin within QIIME2 (Version 2024.10), enabling the generation of high-resolution amplicon sequence variants (ASVs). Taxonomic classification of these ASVs was performed based on the Silva 138.1 reference database, with additional verification via the NCBI taxonomy database. Following normalization procedures, α- and β-diversity metrics were computed to characterize within-sample richness and between-sample community differences. A genus-level abundance table was constructed, from which the 35 most prevalent genera were identified by relative abundance, and hierarchical clustering was applied to visualize microbial distribution patterns across experimental groups.

### 2.10. Statistical Analysis

Descriptive statistics were used to summarize participants’ demographic characteristics, clinical indicators, and body composition data. For continuous variables following a normal distribution, results were reported as mean ± standard deviation (SD). Comparisons between groups were made using either independent sample t-tests or one-way analysis of variance (ANOVA), depending on the context. Categorical data were represented as counts (*n*) and percentages (%), and group differences were analyzed using chi-square tests.

To evaluate microbial diversity, α-diversity was assessed using several indices—including observed species, Chao1, ACE, Shannon, Simpson, and Fisher metrics—via QIIME2 software, which provided insight into within-sample richness and diversity. β-diversity analyses were employed to identify structural differences in microbial communities across samples. Phylogenetic distances, namely unweighted and weighted UniFrac, were computed based on evolutionary relationships among microbial features. These distances were converted into distance matrices and further analyzed using principal coordinates analysis (PCoA), which projects the data onto orthogonal axes, with the first few coordinates capturing the greatest variance. Additionally, the Mann–Whitney U test was used to detect significant taxonomic changes (from phylum to species levels) before and after LPT intervention across different aging durations. Associations between changes in metabolic profiles and gut microbiota abundance at the genus level were examined using Spearman correlation analysis. All statistical procedures were performed using R version 4.1.3, with a threshold of *p* < 0.05 considered statistically significant.

## 3. Results

### 3.1. Basic Characteristics of Participants

Table 1 presents the basic demographic information for the overall population as well as for the intervention groups based on different aging durations of LPT in this study. A total of 71 participants were included, comprising 19 males (26.8%) and 52 females (73.2%). No significant differences were found between the different intervention groups with regard to age, gender, marital status, educational level, monthly income, alcohol consumption, and tea-drinking habits.

### 3.2. Impact of LPT on Metabolic Parameters in MetS Patients

Significant reductions in SBP were observed in the 4-, 7-, and 10-year-aged groups compared to baseline (*p* < 0.05), while DBP decreased notably in the 10-year-aged group (*p* < 0.05). Lipid metabolism improvements included reduced TC in the 1-year-aged group, elevated HDL-C in the 4-year- and 7-year-aged groups, and decreased LDL-C across all aging durations (*p* < 0.05). Although FBG levels declined in all groups, the changes were not statistically significant (Table 2). LPT showed minimal effects on liver health biomarkers (T-BIL, ALT, AST, ALP, CK, LDH), indicating no significant impact on liver function and reinforcing its safety profile (Table S1).

### 3.3. Impact of LPT on Body Weight and Body Composition Parameters in MetS Patients

LPT positively regulated body weight and composition in MetS patients (Table 2). Significant reductions in body weight, BFM, BFP, and VFA were observed across all aging durations (*p* < 0.05). Increases in LBM and MM were noted, with significant changes in the 1-year-aged group (*p* < 0.05). BMI and WC also decreased significantly in the 4-, 7-, and 10-year-aged groups (*p* < 0.05). Based on a sample size of 71, a two-sided test with a significance level of 0.05, the post hoc power analysis for body weight as the outcome showed a power of 98.6%.

### 3.4. Comparison of LPT Interventions with Different Aging Times on Metabolic and Physical Parameters

No significant differences were found in blood pressure (SBP, DBP), lipid metabolism (TC, TG, HDL-C, LDL-C), glycometabolism (HbA1c, FBG), or body composition (weight, BFM, BMI, BFP) among LPT with different aging durations, as all intergroup *p*-values exceeded 0.05 (Table S2). In addition, no significant changes were observed in the subjects’ appetite scores before and after the intervention (Table S3).

### 3.5. Effects of LPT with Different Aging Years on the Gut Microbiota of MetS Patients

**Figure 2:**
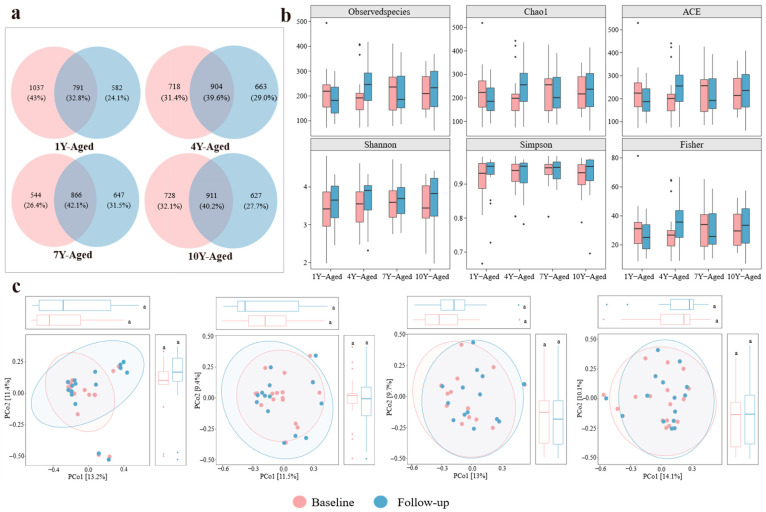
Analysis of alpha diversity and microbial community structure in gut microbiota across four LPT aging groups. (**a**) Venn diagram illustrating the number of unique and shared amplicon sequence variants among different LPT aging durations. (**b**) Boxplots presenting alpha diversity indices including observed species, Chao1, ACE, Shannon, Simpson, and Fisher metrics across the four groups. (**c**) Principal coordinate analysis depicting variations in gut microbial community composition among the 1-year-, 4-year-, 7-year-, and 10-year-aged groups before and after intervention. The letter “a” is employed as part of the letter labeling system to denote statistical significance. Specifically, values sharing the same letter indicate no significant difference at the 0.05 level (*p* > 0.05), whereas those bearing different letters indicate a statistically significant difference (*p* < 0.05).

The Venn diagram illustrated the shared and unique ASVs among samples in different LPT aging groups (Figure 2a). The 7-year-aged group exhibited an increase in the number of unique ASVs after intervention, whereas other groups showed a decrease. The α-diversity of gut microbiota in the four groups was measured using the Observed Species, Chao1, ACE, Shannon, Simpson, and Fisher indices (Figure 2b). The results indicated no significant differences in microbial diversity before and after intervention across the 1, 4-, 7-, and 10-year-aged groups. PCoA revealed no significant differences in the gut microbiota structure of the four aging groups (Figure 2c).

**Figure 3:**
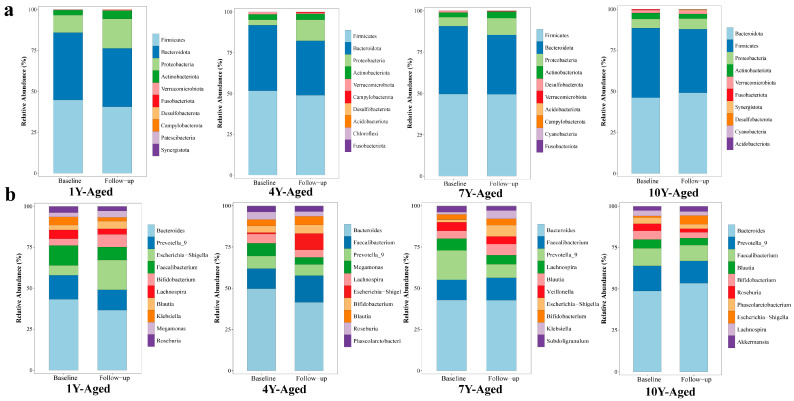
Changes in the relative abundances of gut microbiota at the phylum (**a**) and genus (**b**) taxonomic levels, comparing the periods before and after intervention with LPT of different aging durations.

At the phylum level, analysis revealed that the gut microbiota of patients with MetS was predominantly composed of five phyla: Firmicutes, Bacteroidota, Proteobacteria, Actinobacteriota, and Verrucomicrobiota (Figure 3a). Firmicutes and Bacteroidota were the dominant phyla, accounting for over 80% of the gut microbiota in these patients. After LPT intervention, the relative abundances of Firmicutes decreased in the 1-year-, 4-year-, and 10-year-aged groups, while Bacteroidota abundance decreased in the 1-year-, 4-year-, and 7-year-aged groups. In the 10-year-aged group, the relative abundance of Bacteroidota increased, and the Firmicutes/Bacteroidota ratio decreased. Across all intervention groups, the species abundances of Proteobacteria in MetS patients exhibited an upward trend. At the genus level, the overall gut microbiota profiles exhibited a comparable structure among the four intervention groups (Figure 3b). Bacteroides emerged as the most abundant genus in all groups aged 1, 4, 7, and 10 years, followed by Prevotella_9 and Faecalibacterium. In the 1-year-aged group, notable increases in Escherichia−Shigella and Bifidobacterium were observed relative to baseline values.

**Figure 4:**
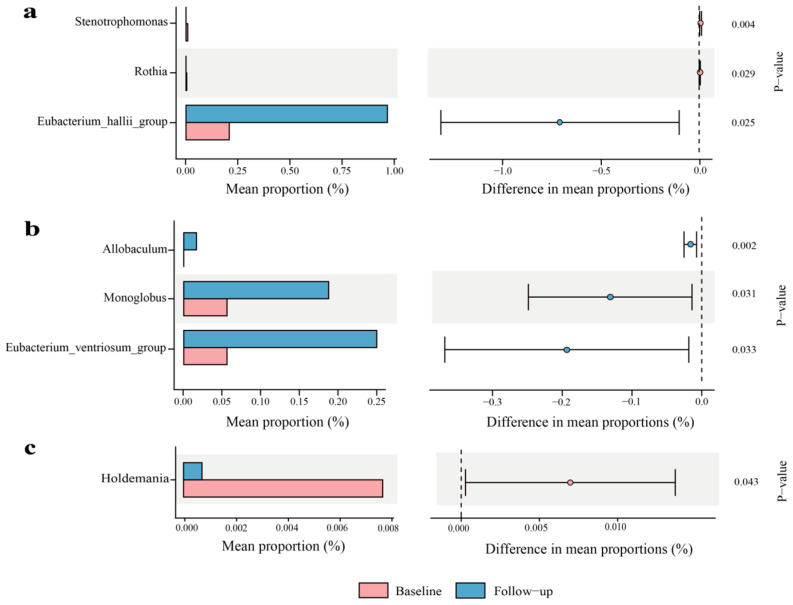
Genus-level analysis of gut bacteria in LPT groups with different aging durations (only statistically significant results are shown): (**a**) 1-year-aged group; (**b**) 4-year-aged group; (**c**) 7-year-aged group.

Microbial community structures were significantly altered post-intervention in the 1-, 4-, and 7-year-aged groups, whereas the 10-year-aged group showed no significant shift. Specifically, the 1-year-aged group experienced marked reductions in Stenotrophomonas and Rothia, alongside an elevation in Eubacterium_hallii_group (Figure 4a). For the 4-year-aged group, Allobaculum, Monoglobus, and Eubacterium_ventriosum_group exhibited significant enrichment (Figure 4b). A significant reduction in Holdemania was detected in the 7-year-aged group (Figure 4c).

**Figure 5:**
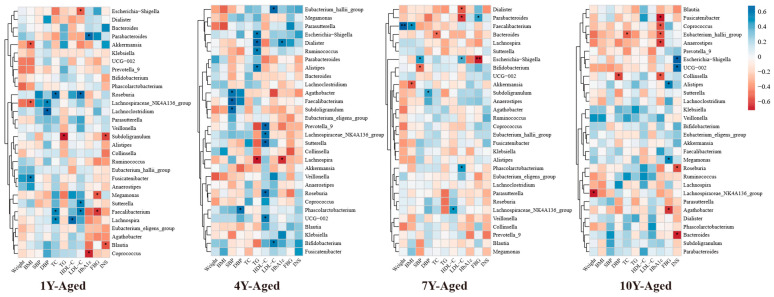
Heat map of correlation analysis between changes in metabolic indicators and changes in intestinal flora (at the genus level) in the LPT group with different aged years. * *p* < 0.05, ** *p* < 0.001.

Further insights from Spearman correlation analysis showed distinct patterns of association between genus-level microbial changes and metabolic indicators across different aging groups (Figure 5). For instance, Akkermansia displayed a strong inverse relationship with BMI in the 1-year- and 7-year-aged groups. In the 1-year-aged group, Subdoligranulum was significantly negatively correlated with both TG and INS levels. Meanwhile, Lachnospiraceae_NK4A136_group demonstrated a significant positive association with HDL-C in both the 4-year- and 10-year-aged groups, and a negative correlation with body weight in the 10-year-aged group.

## 4. Discussion

### 4.1. Major Findings and Comparison with Previous Research

In this study, a randomized, double-blind, longitudinal trial was conducted to evaluate the regulatory effects of LPT on multidimensional metabolic parameters and its impact on the gut microbiota in patients with MetS.

The blood-pressure-lowering effect of LPT observed in this study aligns with findings from previous research on black tea. A systematic review by Liu et al. noted that black tea consumption has a protective effect on SBP [23]. In an intervention study, Hodgson et al. demonstrated that regular consumption of three cups of black tea daily for 6 months significantly reduced SBP and DBP [24]. Similarly, studies on pu-erh tea, a type of dark tea, by Luo et al. indicated that aqueous extracts of pu-erh tea induce endothelium-independent vasodilation of the thoracic aorta and may reduce vascular contractility by inhibiting extracellular Ca^2+^ influx into smooth muscle cells, thereby exerting vasodilatory activity [25].

Tian et al.’s systematic review on tea consumption in MetS patients found that significant improvements in body weight, BMI, and levels of HDL-C, LDL-C, TG, and TC [26]. Huang et al. demonstrated that liquid pu-erh tea extract reduces TC, TG, and LDL-C levels, while effectively inhibiting the decrease in serum HDL-C levels [27]. Wu et al. further confirmed through in vitro models that LPT extract significantly lowers TC, TG, and LDL-C levels while increasing HDL-C levels [19]. Additionally, Ding et al.’s study showed that LPT extract significantly alleviates hyperglycemia, insulin resistance, and blood lipid levels, with high-dose LPT extract exhibiting comparable hypoglycemic effects to metformin [28]. In contrast, the present study observed significant reductions in TC, LDL-C, and HDL-C, but not in TG or FBG, as reported in in vitro investigations. This divergence may be attributed to two primary factors: First, the bioavailability of active constituents differs substantially between in vitro cellular systems and the human in vivo milieu, where intestinal metabolism and hepatic first-pass effects likely attenuate their systemic exposure. Second, the ethanol extraction protocol employed in prior in vitro studies enables efficient solubilization of polar bioactive compounds, whereas the aqueous brewing method used herein may limit the release of lipophilic components, potentially resulting in suboptimal effective dosages.

This study also reports the positive effects of LPT on reducing body weight, BMI, BFM, and BFP in MetS patients. Basu et al. found that green tea beverage or extract interventions for 8 weeks significantly reduced body weight and BMI [29]. In a randomized controlled trial with a similar intervention duration to this study, Yang et al. observed that male MetS patients experienced a significant weight loss of approximately 1.3 kg and a reduction in BMI after taking pu-erh tea extract capsules [30]. Interestingly, despite the same intervention duration, the comparable weight loss effect achieved by LPT through water brewing in this study is equivalent to that of pu-erh tea extract capsules, indicating that LPT has greater advantages in metabolic regulatory activity without relying on complex component purification processes.

Notably, Fuzhuan brick tea (FBT), another post-fermented dark tea, has been shown to significantly alleviate MetS components in mice through its polysaccharide content [31]. Hou et al. demonstrated that pu-erh tea improves liver, jejunum, and adipose tissue function in MetS mouse models by regulating circadian rhythm pathways, upregulating hepatic antioxidant levels, and downregulating inflammatory cytokines [32]. Wei et al. isolated a natural polysaccharide (TPS-5) from LPT, which exhibits dose-dependent inhibitory effects on α-amylase and α-glucosidase, along with free radical scavenging, bile salt-binding, hypolipidemic, and hypoglycemic activities [33]. Zhou et al.’s multi-omics study identified ellagic acid, catechins, and naringenin as key active components of LPT for alleviating hyperlipidemia [34]. This study observed that LPT improves glycolipid metabolism in MetS patients, suggesting that LPT may exert metabolic regulatory effects through a similar polysaccharide–polyphenol complex mechanism.

In the 10-year-aged group of this study, a decrease in the Firmicutes/Bacteroides (F/B) ratio was observed. This finding is consistent with Zhou et al., who found that dark tea extracts helped protect the intestinal barrier and reduced the F/B ratio when comparing the regulatory effects of six different tea extracts on the intestinal microbiota of HFD-induced MetS rats [35]. Similarly, Yue et al. found that theabrownin isolated from pu-erh tea could reduce the F/B ratio in MetS rats in a dose-dependent manner [36]. In a study on the protective effect of LPT on HFD/cold-exposure-induced irritable bowel syndrome in rats, LPT significantly reduced the F/B ratio and reconstructed the microbial pattern, alleviating IBS by repairing gastrointestinal dysfunction, regulating pro-inflammatory cytokine secretion, and restoring microbial homeostasis [37].

Significant decreases in the abundances of Stenotrophomonas and Rothia were observed in the 1-year-aged group of this study. Stenotrophomonas is a human opportunistic pathogen that has become an important pathogen of concern in immunocompromised patient populations [38]. The reduction in these two genera helps alleviate systemic inflammatory status [39]. Following LPT intervention, this study found enrichment of the short-chain fatty acid (SCFA)-producing bacterium Eubacterium_hallii_group. The Eubacterium_hallii_group is a critical intestinal microbial community capable of fermenting dietary fiber to produce SCFAs, particularly butyrate, which confers multiple benefits to host health. Butyrate activates GPR109A receptors on intestinal epithelial cells to inhibit the NF-κB signaling pathway, thereby reducing the release of pro-inflammatory factors and improving insulin sensitivity and vascular tone [40,41].

In the 1-year- and 7-year-aged groups, a significant negative correlation was observed between BMI and the abundance of Akkermansia. As a well-recognized beneficial gut bacterium, Akkermansia modulates host metabolism by maintaining intestinal barrier integrity and participating in SCFA metabolism. Elevated abundance of Akkermansia is commonly associated with improved obesity metrics [42]. Subdoligranulum, a potential SCFA-producing bacterium, may reduce triglyceride levels and enhance insulin sensitivity through mechanisms such as inhibiting fat synthesis and promoting glycolysis when its abundance increases [43]. The enrichment of Lachnospiraceae_NK4A136_group may influence lipid profiles by participating in reverse cholesterol transport or regulating bile acid metabolism, with its role in energy metabolism regulation becoming increasingly prominent in the 10-year-aged group [44]. These findings suggest that LPT aged for different durations exerts targeted effects on metabolic indicators by shaping distinct gut microbiota compositions.

Interestingly, while Chinese folklore holds that LPT improves with age in terms of taste, this study found no significant differences among 1-year, 4-year, 7-year, and 10-year-aged LPT groups in regulating glycometabolism, lipid metabolism, and body composition in MetS patients from a health efficacy perspective. Regarding flavor characteristics, Liang et al. found that the long-term storage of LPT enhances its flavor and commercial value, particularly when stored for over 9 years [45]. Huang et al.’s study also indicated that the optimal storage period for LPT is 8–10 years [46]. However, in this study, with the increase in aging years, components such as tea polyphenols and catechins gradually decreased, theabrownin accumulated continuously, flavonoids showed a trend of first increasing and then decreasing, and soluble dietary fiber increased significantly in the later stage. Therefore, although storage time has a clear impact on the chemical composition and flavor of Liupao tea, its effect on human metabolic health may not be solely regulated by aging time. This suggests that future studies need to further analyze the correlation mechanism between component changes and health effects, rather than simply relying on aging time to predict efficacy.

### 4.2. The Limitations of the Study

This study has certain limitations that should be acknowledged. First, the lack of a dedicated control group—since all participants received LPT intervention across varying aging durations—makes it challenging to rule out potential time-related effects or other confounding variables. As a result, the independent impact of LPT on health outcomes cannot be definitively determined, and the observed metabolic improvements may not be entirely attributable to LPT consumption alone. Second, the study did not include systematic tracking of participants’ dietary intake or physical activity. Although individuals were advised to maintain their habitual lifestyles, the absence of objective, quantitative assessments leaves room for potential variation in these behaviors, which may have influenced the intervention outcomes. Together, these limitations may compromise the internal validity and limit the generalizability of the findings to broader populations.

### 4.3. Practical Implications and Future Directions

The findings of this study highlight the potential of LPT as a functional beverage for managing MetS. Its effects in improving blood pressure, lipid profiles, body composition, and gut microbiota indicate that LPT could serve as an adjuvant dietary intervention for individuals with MetS. Given its high accessibility, LPT may offer a cost-effective and natural approach to metabolic health management. Future research should include a control group and explore changes in diet and physical activity using dietary recall methods and wearable devices, to better differentiate the specific effects of LPT. Additionally, the intervention duration will be extended to evaluate the sustainability of metabolic improvements. Mechanistic studies such as metabolomics will also be conducted to clarify how LPT modulates metabolic pathways, providing guidance for its clinical application in MetS management.

## 5. Conclusions

LPT with different aging years has significant effects in improving blood pressure, and blood lipids in patients with MetS. Patients’ body weight and body composition parameters have been significantly improved, and changes in gut microbiota have been observed. No significant differences were observed in the improvement of metabolic health among LPT with different aging years.

## Figures and Tables

**Table 1:** The basic demographic information of the study subjects ^a^.

Characteristic	Total Population Group (*n* = 71)	1-Year-Aged Group (*n* = 19)	4-Year-Aged Group (*n* = 17)	7-Year-Aged Group (*n* = 18)	10-Year-Aged Group (*n* = 17)	*p*
Age (years), mean ± SD	53.5 ± 16.3	53.6 ±17.8	55.2 ±14.2	50.5 ± 18.7	54.9 ± 14.7	0.825
Gender, *n* (%)						0.901
Male	19 (26.8)	5 (26.3)	4 (23.5)	6 (33.3)	4 (23.5)	
Female	52 (73.2)	14 (73.7)	13 (76.3)	12 (66.7)	13 (76.5)	
Marital Status, *n* (%)						0.501
Married/Cohabiting	56 (78.9)	15 (78.9)	15 (88.2)	12 (66.7)	14 (82.4)	
Single/Divorced/Widowed	15 (21.1)	4 (21.1)	2 (11.8)	6 (33.3)	3 (17.6)	
Educational Level, *n* (%)						0.935
Junior high school and below	5 (7.0)	1 (5.3)	1 (5.9)	2 (11.1)	1 (5.9)	
High school	11 (15.5))	3 (15.8)	2 (11.8)	2 (11.1)	4 (23.5)	
Bachelor’s degree and above	55 (77.5)	15 (78.9)	14 (82.4)	14 (77.8)	12 (70.6)	
Average Monthly Income, *n* (%)						0.324
RMB < 3000	9 (12.6)	3 (15.8)	0 (0.0)	4 (22.2)	2 (11.8)	
RMB 3000~8000	31 (43.7)	8 (42.1)	9 (52.9)	4 (22.2)	10 (58.8)	
RMB ≥ 8000	31 (43.7)	8 (42.1)	8 (47.1)	10 (55.6)	5 (29.4)	
Smoking, *n* (%)						0.119
Never smoked	66 (93.0)	17 (89.5)	14 (82.4)	18 (100.0)	17 (100.0)	
Former smoker	3 (4.2)	2 (10.5)	1 (5.9)	0 (0.0)	0 (0.0)	
Current smoker	2 (2.8)	0 (0.0)	2 (11.8)	0 (0.0)	0 (0.0)	
Alcohol Consumption, *n* (%)						0.179
Never drinks	63 (88.7)	18 (94.7)	13 (76.5)	16 (88.9)	16 (94.1)	
Former drinker	6 (8.5)	1 (5.3)	4 (23.5)	1 (5.6)	0 (0.0)	
Current drinker	2 (2.8)	0 (0.0)	0 (0.0)	1 (5.6)	1 (5.9)	
Frequent Tea Drinking, *n*(%)						0.411
Yes	33 (46.5)	9 (47.4)	9 (52.9)	10 (55.6)	5 (29.4)	
No	38 (53.5)	10 (52.6)	8 (47.1)	8 (44.4)	12 (70.6)	

^a^ Continuous variables are presented as mean ± SD; categorical variables are shown as frequencies (*n*) and percentages (%); SD, standard deviation; RMB, Renminbi.

**Table 2:** Impact of LPT treatment on metabolic profiles and body composition in MetS subjects.

Parameters	1-Year-Aged Group (*n* = 19)		4-Year-Aged Group (*n* = 17)		7-Year-Aged Group (*n* = 18)		10-Year-Aged Group (*n* = 17)	
	Baseline	Follow-Up	Baseline	Follow-Up	Baseline	Follow-Up	Baseline	Follow-Up
Metabolic parameters								
SBP, mmHg	128.58 ± 22.29	124.00 ± 18.69	129.06 ± 15.43	120.76 ± 17.64 *	134.11 ± 13.63	116.34 ± 27.12 *	132.41 ± 16.53	124.41 ± 15.78 *
DBP, mmHg	74.95 ± 12.99	72.79 ± 12.21	77.65 ± 9.77	73.88 ± 10.16	79.72 ± 10.47	75.83 ± 17.44	85.24 ± 15.47	75.35 ± 10.70 *
TC, mmol/L	5.43 ± 1.03	5.11 ± 0.85 *	5.51 ± 1.09	5.36 ± 1.03	5.44 ± 0.90	5.57 ± 0.86	5.60 ± 0.70	5.15 ± 0.88
TG, mmol/L	1.34 ± 0.92	1.37 ± 1.00	1.51 ± 1.31	1.21 ± 0.58	1.82 ± 0.92	1.87 ± 1.26	2.10 ± 2.17	1.88 ± 1.88
HDL-C, mmol/L	1.36 ± 0.41	1.39 ± 0.34	1.28 ± 0.22	1.40 ± 0.21 *	1.29 ± 0.31	1.38 ± 0.26 *	1.34 ± 0.26	1.37 ± 0.18
LDL-C, mmol/L	3.13 ± 0.62	2.77 ± 0.54 **	3.29 ± 0.80	3.03 ± 0.72 *	3.28 ± 0.60	3.02 ± 0.54 *	3.15 ± 0.47	2.73 ± 0.59 *
APOA1, g/L	1.34 ± 0.26	1.26 ± 0.22 *	1.31 ± 0.16	1.26 ± 0.12	1.31 ± 0.16	1.24 ± 0.12 *	1.37 ± 0.17	1.26 ± 0.13 **
APOB, g/L	0.90 ± 0.15	0.86 ± 0.14 *	0.94 ± 0.19	0.93 ± 0.24	0.94 ± 0.14	0.89 ± 0.12	0.88 ± 0.15	0.85 ± 0.15
HbA1c, %	5.58 ± 1.07	5.65 ± 1.07	5.74 ± 0.76	5.74 ± 0.75	6.04 ± 1.34	6.03 ± 1.20	6.01 ± 1.51	6.08 ± 1.62
FBG, mmol/L	5.90 ± 2.06	5.82 ± 2.27	5.89 ± 1.35	5.70 ± 1.16	9.43 ± 13.26	6.28 ± 1.86	6.49 ± 2.90	6.28 ± 2.30
INS, pmol/L	71.11 ± 35.14	71.25 ± 48.32	67.57 ± 28.76	61.07 ± 30.41	74.24 ± 29.98	72.88 ± 37.20	92.59 ± 58.33	80.92 ± 52.34
Body composition								
Weight, kg	66.82 ± 13.10	65.68 ± 13.16 *	66.42 ± 7.18	64.60 ± 7.07 *	68.83 ± 10.70	66.26 ± 11.29 *	69.75 ± 10.13	68.15 ± 11.60 *
BFM, kg	21.27 ± 5.01	19.92 ± 4.72 **	21.48 ± 4.11	19.82 ± 4.07 **	21.25 ± 4.78	19.11 ± 4.27 **	22.09 ± 6.08	20.12 ± 5.43 **
LBM, kg	45.87 ± 10.01	46.50 ± 10.64	44.95 ± 5.22	45.32 ± 5.72	47.56 ± 8.34	47.73 ± 9.13	46.66 ± 7.77	47.17 ± 8.49
MM, kg	42.06 ± 9.30	42.71 ± 9.92 *	41.17 ± 4.88	41.03 ± 5.51	43.65 ± 7.77	43.91 ± 8.52	42.71 ± 7.23	43.26 ± 7.90
BMI, kg/m^2^	25.28 ± 2.96	24.95 ± 3.07	25.81 ± 2.51	25.13 ± 2.46 *	25.36 ± 2.72	24.48 ± 2.85 *	26.60 ± 2.93	26.03 ± 3.24 *
WC, cm	83.95 ± 8.35	82.24 ± 7.54 **	84.26 ± 6.45	81.45 ± 5.78 *	85.37 ± 8.12	82.69 ± 7.81 **	85.92 ± 7.79	79.34 ± 20.64
BFP, %	31.24 ± 5.42	30.11 ± 4.96 *	32.28 ± 4.63	30.38 ± 4.89 *	30.87 ± 5.37	28.66 ± 4.99 **	33.28 ± 5.05	31.31 ± 5.11 **
VFA, cm^2^	98.72 ± 35.66	87.78 ± 33.37 **	104.47 ± 36.07	84.76 ± 31.59 *	95.50 ± 30.15	80.28 ± 27.30 **	109.5 ± 34.77	93.75 ± 31.89 **

** *p* < 0.001, * *p* < 0.05.

## Data Availability

Due to ethical restrictions and privacy considerations, the datasets generated during this study are not publicly available. However, de-identified data may be obtained from the corresponding author upon reasonable request and approval from the institutional review board.

## Supplementary Materials

The following supporting information can be downloaded at: https://www.mdpi.com/article/10.3390/nu17142371/s1, Figure S1: Proportions of main water-soluble components in Liupao tea with different aging periods; Table S1: The effects of Liupao tea intervention on liver health biomarkers; Table S2: Differences in the effects of Liupao tea with different aging years on improving metabolic parameters and body composition; Table S3: Changes in appetite of subjects before and after intervention with Liupao tea of different aging years.

## Abbreviations

APOA1: Apolipoprotein A1; APOB: Apolipoprotein B; BMI: Body Mass Index; BFP: Body Fat Percentage; BFM: Body Fat Mass; DBP: Diastolic Blood Pressure; FBG: Fasting Blood Glucose; HbA1: Hemoglobin A1c; HDL-C: High-Density Lipoprotein Cholesterol; HFD: High-Fat Diet; INS: Insulin; LBM: Lean Body Mass; LDL-C: Low-Density Lipoprotein Cholesterol; LPT: Liupao Tea; MM: Muscle Mass; MetS: Metabolic Syndrome; SBP: Systolic Blood Pressure; TC: Total Cholesterol; TG: Triglycerides; VFA: Visceral Fat Area; WC: Waist Circumference.
