# Supplementary material for: A Randomized Double-Blind Trial of the Effect of Liupao Tea on Metabolic Parameters, Body Composition, and Gut Microbiota in Adults with Metabolic Syndrome
Source: Nutrients. 2025 Jul 19;17(14):2371. doi: 10.3390/nu17142371 (PMC12300092; doi:10.3390/nu17142371)
Supplement: Supplementary file 1 [file nutrients-17-02371-s001.zip › nutrients-3723701-supplementary.pdf]

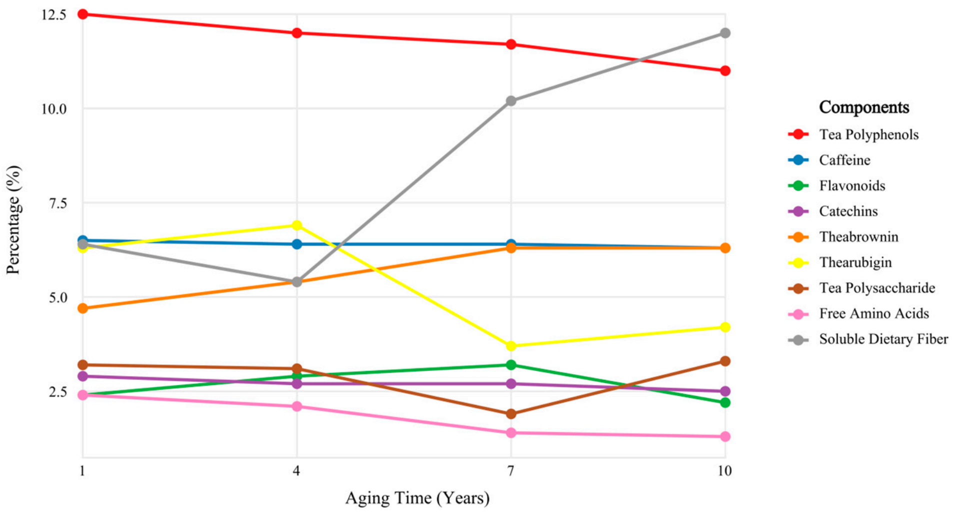

Figure S1. Proportions of main water-soluble components in Liupao tea with different aging periods.

Table S1 The effects of Liupao tea intervention on liver health biomarkers[a]

liver health biomarkers	1-Year Aged Group (n=19)			4-Year Aged Group (n=17)			7-Year Aged Group (n=18)			10-Year Aged Group (n=17)		
	Baseline	Follow-up	*P*	Baseline	Follow-up	*P*	Baseline	Follow-up	*P*	Baseline	Follow-up	*P*
T-BIL, μmol/L	15.29±4.40	14.25±4.91	0.148	13.29±5.41	13.86±4.09	0.470	13.24±3.02	13.30±3.19	0.938	14.58±6.31	14.53±4.99	0.961
ALT, U/L	21.56±9.75	18.94±8.00	0.131	19.62±8.09	17.52±6.96	0.175	27.22±29.21	38.32±47.45	0.203	21.96±9.03	26.07±20.69	0.352
AST, U/L	25.74±8.05	23.05±6.32	0.058	21.76±4.52	20.12±4.00	0.101	25.72±16.01	29.11±20.75	0.406	20.65±5.02	20.29±6.72	0.765
ALP, U/L	70.53±21.41	73.42±21.36	0.240	69.82±16.95	70.06±19.36	0.918	82.89±25.42	78.50±20.59	0.264	76.94±17.7	74.82±17.73	0.301
CK, U/L	90.42±64.05	106.89±70.13	0.027	84.35±38.77	94.06±32.83	0.215	119.28±118.34	122.72±46.69	0.902	87.59±31.42	90.65±37.58	0.700
LDH, U/L	173.42±30.00	177.32±26.95	0.179	167.65±21.66	178.59±36.26	0.062	167.44±31.85	178.5±22.31	0.020	179.18±26.51	181.76±24.34	0.568

[a]T-BIL: total bilirubin; ALT: alanine aminotransferase; AST: aspartate aminotransferase; ALP: serum alkaline phosphatase; CK: creatine kinase; LDH: lactate dehydrogenase.

Table S2 Differences in the effects of Liupao tea with different aging years on improving metabolic parameters and body composition.

Parameters[a]	1 year-aged group (n=19)	4 year-aged group (n=17)	7 year-aged group (n=18)	10 year-aged group (n=17)	*P*
**Metabolic parameters**					
SBP, mmHg	-4.58±10.37	-8.29±8.33	-17.77±26.60	-8.00±11.27	0.086
DBP, mmHg	-2.16±6.88	-3.76±9.88	-3.89±15.20	-9.88±13.58	0.235
TC, mmol/L	-0.32±0.48	-0.15±0.58	0.13±0.88	-0.45±0.91	0.113
TG, mmol/L	0.03±0.37	-0.3±1.22	0.05±0.58	-0.21±0.59	0.420
HDL-C, mmol/L	0.03±0.16	0.12±0.21	0.09±0.13	0.03±0.14	0.233
LDL-C, mmol/L	-0.36±0.34	-0.27±0.34	-0.26±0.34	-0.42±0.60	0.601
APOA1, g/L	-0.08±0.11	-0.05±0.13	-0.07±0.11	-0.11±0.08	0.439
APOB, g/L	-0.05±0.07	-0.01±0.13	-0.05±0.11	-0.03±0.16	0.741
HbA1c, %	0.07±0.19	0.01±0.28	-0.01±0.47	0.07±0.21	0.813
FBG, mmol/L	-0.07±0.41	-0.19±0.59	-3.15±13.46	-0.21±0.86	0.470
INS, pmol/L	0.14±21.85	-6.50±31.04	-1.35±22.79	-11.67±27.19	0.519
**Body composition**					
Weight, kg	-1.36±2.30	-1.82±2.85	-2.57±3.58	-1.64±2.06	0.591
BFM, kg	-1.34±1.36	-1.65±2.25	-2.14±1.70	-1.77±1.09	0.366
LBM, kg	0.63±1.29	0.37±1.43	0.17±1.27	0.52±1.10	0.738
MM, kg	0.65±1.24	-0.14±3.48	0.26±1.22	0.55±1.05	0.658
BMI, kg/m^2	-0.40±0.91	-0.68±1.17	-0.88±1.30	-0.55±0.81	0.567
WC, cm	-1.71±1.70	-2.81±3.43	-2.68±2.06	-6.58±17.71	0.404
BFP, %	-1.13±2.20	-1.89±2.47	-2.21±1.73	-1.97±1.12	0.388
VFA, cm^2	-10.94±9.88	-19.71±23.08	-15.22±11.97	-15.75±10.23	0.385

[a]Abbreviations: SBP: Systolic blood pressure; DBP: Diastolic blood pressure; TC: Total cholesterol; TG: Triglycerides; HDL-C: High-density lipoprotein cholesterol; LDL-C: Low-density lipoprotein cholesterol; APOA1: Apolipoprotein A1; APOB: Apolipoprotein B; HbA1: Hemoglobin A1c; FBG: Fasting blood glucose; INS: Insulin; BFM: Body Fat Mass; LBM: Lean Body Mass; MM: Muscle Mass; BMI: Body Mass Index; WC: Waist Circumference; BFP: Body Fat Percentage; VFA: Visceral Fat Area.

Table S3 Changes in appetite of subjects before and after intervention with Liupao tea of different aging years[a].

Intervention group	Time points	VAS score	*P*
1 year-aged group	Baseline	307.89±63.00	0.927
	Follow-up	305.84±72.91	
4year-aged group	Baseline	319.59±75.88	0.928
	Follow-up	322.29±97.21	
7 year-aged group	Baseline	299.28±74.29	0.083
	Follow-up	342.11±69.42	
10 year-aged group	Baseline	302.12±66.61	0.903
	Follow-up	305.29±83.68	

[a]Based on the Visual Analogue Scale (VAS), the dietary feelings of patients in the past week were investigated. The dietary status of patients was evaluated mainly from five aspects: hunger before meals, fullness after meals, intensity of appetite, amount of food intake, and mood during meals. The score range for each aspect is 0-100 points, with a total score of 500 points. The higher the score, the better the dietary status of the patient.
